# Muscle Fatigue-Alleviating Effects of a Prescription Composed of Polygonati Rhizoma and Notoginseng Radix et Rhizoma

**DOI:** 10.1155/2020/3963045

**Published:** 2020-06-08

**Authors:** Ya-Qin Yang, Yan-Qin Li, Li-Ping Yu, Xue Li, Jian-Kang Mu, Jin Shang, Wen Gu, Jing-Ping Li, Jie Yu, Xing-Xin Yang

**Affiliations:** College of Pharmaceutical Science, Yunnan University of Chinese Medicine, 1076 Yuhua Road, Kunming 650500, China

## Abstract

Long-term muscle fatigue is a major cause of injury. Drugs/nutrients from herbal medicines that prevent fatigue remain a major research focus. In China, a prescription composed of Polygonati Rhizoma and Notoginseng Radix et Rhizoma has been commonly used as a herb and food nutrient, providing protection against fatigue in the clinic. To date, the mechanisms through which this prescription prevented fatigue are unknown. Here, we identified the effects of this prescription on muscle fatigue based on energy and oxidation regulation. Fatigue mouse models were produced through weight-bearing exhaustive swimming. Mice were intragastrically administered prescription extracts (1 and 2 g/kg) for four weeks. Changes in exhaustive swimming times, antifatigue biochemical indicators, oxidative status, and energy metabolism were investigated. The prescription prolonged the exhaustive swimming time of the mice. The content of lactic acid and blood urea nitrogen in the serum was also markedly reduced by the prescription. The content of liver glycogen and lactate dehydrogenase in the serum increased. The prescription also significantly reduced malondialdehyde levels and increased the levels of superoxide dismutase and glutathione peroxidase. The levels of ATPase, complexes I and II in the mitochondria of hind-leg skeletal muscle, and serum creatine kinase also increased in response to the prescription. Our results indicated that the prescription could effectively alleviate muscle fatigue status by promoting energy metabolism and antioxidation ability. The prescription therefore represents a useful drug/nutrient strategy to alleviate muscle fatigue.

## 1. Introduction

Muscle fatigue is a complex process in which a series of physiological and biochemical changes occur in the body, including energy overconsumption, the production and accumulation of metabolites, dysfunction of the immune system, excessive production of reactive oxygen species, and damage to cellular structures [1]. Muscle fatigue leads to a series of physiological changes, including aches, sleep disorders, endocrine disorders, immune dysfunction, and metabolic disorders. If untreated, long-term muscle fatigue leads to myophagism, depression, aging and other diseases [2]. Developing drugs/nutrients to alleviate fatigue and improve body health represents a major research focus.

In China, a prescription composed of Polygonati Rhizoma (PR) and Notoginseng Radix et Rhizoma (NRR) (PR : NRR = 1 : 1) has been used as an herb and food nutrient and is effective against fatigue and related disease in the clinic. PR was first recorded in *Mingyi Bielu* (A.D. 220-450, written by HongJing Tao) and has been used as a traditional Chinese medicine (TCM) and nutrient for over 2000 years. PR is comprised of saponins and polysaccharides and has pharmacological activity, including antifatigue, immune promotion, antiaging, blood glucose regulation, and lipid regulation [3–6]. NRR has also been used as a TCM and nutrient for over 2000 years. It is comprised of saponins, dencichine, amino acids, and polysaccharides with various pharmacological activities, including antiatherosclerosis, antidiabetes, antifatigue, anticancer, and anticardiovascular disease [7]. This contributes to its wide range of applications in the clinic and for health care.

Despite the known antifatigue effects of the prescription, knowledge of its mechanism of action is lacking. Here, we investigated the muscle fatigue-alleviating effects of the prescription in fatigue mouse models induced by weight-bearing exhaustive swimming. The results indicated that this prescription could improve muscle fatigue status by promoting energy metabolism and antioxidation ability. The prescription therefore represents a useful drug/nutrient supplement to alleviate muscle fatigue.

## 2. Materials and Methods

### 2.1. Chemicals, Reagents, and Materials

Complex I and complex II determination kits were purchased from Beijing Solarbio Science & Technology Co., Ltd. (Beijing, China). Bicinchoninic acid (BCA) protein determination kits were provided by Beyotime Institute of Biotechnology (Shanghai, China). High-purity deionized water was purified using a Milli-Q System (Millipore, Bedford, MA, USA). Lactic acid (LA), blood urea nitrogen (BUN), malondialdehyde (MDA), superoxide dismutase (SOD), glutathione peroxidase (GSH-PX), creatine kinase (CK), lactate dehydrogenase (LDH), liver glycogen (LG), and ATP synthase (ATPase) determination kits were purchased from Nanjing Jiancheng Bioengineering Research Institute (Nanjing, China). All other reagents were of analytical grade or higher. Panacis Quinquefolii Radix (PQR) powder was provided by Zhongzhi Yinpian Co., Ltd. (Guangdong, China). PR and NRR (purchase date August 16, 2019) were purchased from the Wenshan Shengnong Trueborn Medicinal Materials Cultivation Cooperation Society (Wenshan, China). Samples were authenticated by Professor Jie Yu, and a voucher specimen of PR (No. 9524) and NRR (No. 9525) was deposited in the Key Laboratory of Preventing Metabolic Diseases of Traditional Chinese Medicine, Yunnan University of Chinese Medicine (Kunming, China).

### 2.2. Preparation of the Prescription Extract

Fresh PR was processed as previously described [3]. Briefly, fresh rhizome was separated from fibrous roots, washed, cut into thick slices, and dried in a dryer (YHG-S, Shanghai Yuejin Medical Instrument Factory, Shanghai, China) at 50°C. Dried materials were infiltrated in a 5-fold volume of Shaoxing Rice Wine (Beijing Ershang Wangzhihe Food Co., Ltd., Beijing, China) and steamed in a steam sterilizer (LDZX-50 KBS, Shanghai Shenan Medical Instrument Factory, Shanghai, China) for 2.5 h at 120°C. The steamed materials were dried at 60°C.

Next, steam dried PR and dried NRR were pulverized and mixed at a 1 : 1 ratio. The mixed powder was immersed in a 10-fold volume of water for 30 min and decocted with water for 60 min. Filtrates were collected after leaching. Dregs were successively decocted with ten volumes of water for 60 min, and liquids were filtered. Two successive filtrates were combined and condensed using an N-1100D-WD rotatory evaporator (Ai Lang Instrument Co., Ltd., Shanghai, China) under reduced pressure at 50°C. Finally, concentrates were lyophilized to powder using a FD5-3 freeze dryer (SIM International Group Co. Ltd., Newark, DE, USA). The obtained powder was stored at room temperature in a desiccator.

### 2.3. Animals and Experimental Design

All procedures involving animals complied with the Guide for the Care and Use of Laboratory Animals as published by the US National Institutes of Health. Protocols were approved by the Institutional Ethical Committee on Animal Care and Experimentations of Yunnan University of Chinese Medicine (Kunming, China) (R-062019S003).

Healthy male Kunming mice (38 ± 2 g) were provided by Liaoning Changsheng Biotechnology Co., Ltd. (Liaoning, China). Mice were kept in a room controlled by temperature (24 ± 1°C) and humidity (65 ± 10%) in a 12-hour light/dark cycle (light from 08:00 am. to 08:00 pm). Food and water were provided ad libitum. After one week of adaptive feeding, mice were randomly divided into the following five groups: (1) normal control group (normal saline), (2) model group (normal saline), (3) PQR group (0.6 g/kg), (4) low-dose prescription (LP) group (1 g/kg), and (5) high-dose prescription (HP) group (2 g/kg). Mice were intragastrically administered with the corresponding test samples (or normal saline) once per day for 30 days. PQR powder and prescription extracts were separately prepared in normal saline.

### 2.4. Sample Collection

Thirty minutes after the final administration on day 30, excluding the normal control group, the tails of the mice were bare with a lead sheath 5% of the mouse weight, and mice were placed into a water tank with a water temperature of 25 ± 1°C to perform swimming. Swimming times were recorded until the mice sank to the bottom for 8 seconds. Mice were removed from the water and wiped with a paper towel for drying. The left eyeball was removed to collect blood samples which were allowed to clot at 4°C. Samples were centrifuged at 3500 rpm for 15 min. Finally, the mouse was sacrificed by cervical dislocation, and liver and skeletal muscles of the hind legs were harvested. All samples were stored at -80°C until use.

### 2.5. Determination of Biochemical Parameters of Serum and Liver

Serum LA, LDH, and BUN were measured. Liver homogenates and supernatants (protein concentrations determined by BCA assays) were used to determine the levels of LG, MDA, SOD, and GSH-PX. All parameters were measured on a SpectraMax Plus 384 Microplate Reader (Molecular Devices, Sunnyvale, CA, USA) using commercially available diagnostic kits, in accordance with the manufacturer's instructions.

### 2.6. Evaluation of Energy Metabolism-Related Indicators

The mitochondria of the skeletal muscles of the hind legs of mice were isolated as previously described [8]. Briefly, skeletal muscle (0.1 g) samples were placed into an ice-cold isolation buffer (210 mM mannitol, 70 mM sucrose, 10 mM Tris base, 1 mM EDTA, and 0.5 mM EGTA, pH 7.4) to remove blood, and samples were minced into 1 mm^3^ and homogenized in the isolation buffer. After centrifugation at 1,000 × g for 10 min, the supernatants were collected and centrifuged at 10,000 × g for 10 min. Precipitates were resuspended in the isolation buffer and centrifuged at 10,000 × g for 10 min to obtain mitochondria. All procedures were performed on ice or at 4°C in the cold room.

Next, the mitochondria were resuspended in normal saline. After determining the protein concentrations by BCA assay, 100 *μ*L of mitochondrial suspensions was used for the determination of ATPase, complex I, and complex II levels using commercially available diagnostic kits. Values were measured on a SpectraMax Plus 384 Microplate Reader (Molecular Devices, Sunnyvale, CA, USA).

### 2.7. Statistical Processing

Data were expressed as the mean ± standard deviation (S.D.). *P* < 0.05 or *P* < 0.01, as tested by a one-way analysis of variance, were deemed significant differences. Differences between two groups were analyzed by two-tailed Student's *t*-test. SPSS version 21.0 was used for all statistical analyses (IBM, Armonk, NY, USA).

## 3. Results

### 3.1. Effects of Prescription on Body Weight and Food Intake

The prescription extract was administered by oral gavage at dosages of either 1 or 2 g/kg/day for 30 days. During the study, none of the mice died and they had healthy-looking fur, normal drinking habits, moved freely, and rapidly responded to external stimuli. As shown in Table 1, there was no significant difference in body weight and food intake among the groups during treatment. These suggested unharmful effects were caused by the prolonged treatment.

### 3.2. Effects of Prescription on Exhaustive Swimming Time in Mice

**Figure 1:**
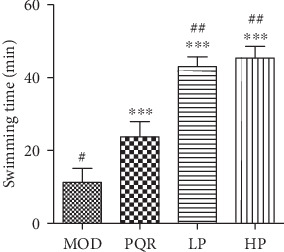
Effects of the prescription on exhaustive swimming times in mice. Mice were administered saline, PQR, or the prescription extract (1 or 2 g/kg/day). After 4 weeks of feeding, mice performed the weight-bearing exhaustive swimming test, and swimming times were recorded. Values represent the mean ± S.D. from 7 animals. ^∗^*P* < 0.05, ^∗∗^*P* < 0.01, and ^∗∗∗^*P* < 0.001 versus the relative MOD group. ^#^*P* < 0.05, ^##^*P* < 0.01, and ^###^*P* < 0.001 versus the relative PQR group. HP: high-dose prescription; LP: low-dose prescription; MOD: model; PQR: Panacis Quinquefolii Radix.

We used the experimental model of weight-bearing exhaustive swimming in mice to study the effects of the prescription on muscle fatigue status. We found that swimming times were significantly prolonged after treatment with the prescription and that the swimming time of the prescription group was significantly longer than that of the positive control group (Figure 1).

### 3.3. Effects of the Prescription on Antifatigue Biochemical Indicators in Mice

**Figure 2:**
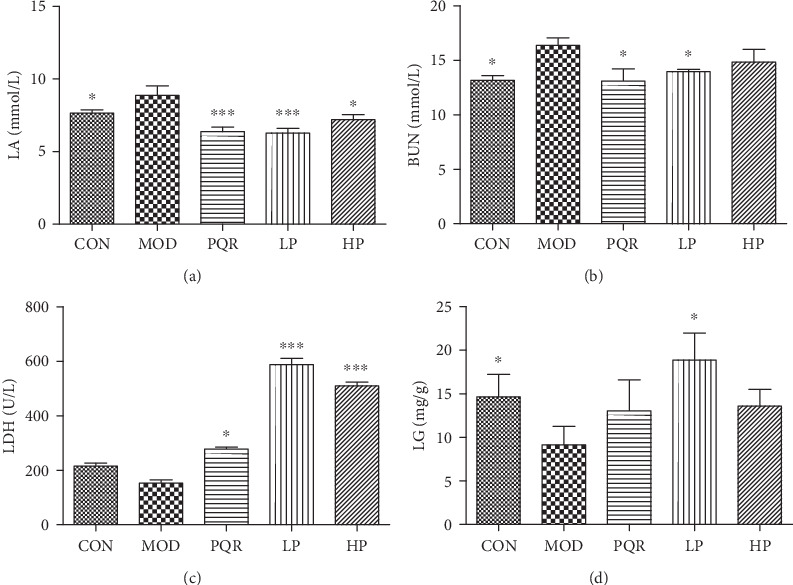
Effects of the prescription on antifatigue biochemical indicators in mice. Mice were administered either saline, PQR, or the prescription extract (1 or 2 g/kg/day). After 4 weeks of feeding, mice were sacrificed and serum and liver samples were collected. Levels of (a) LA, (b) BUN, and (c) LDH in the serum and (d) LG in the liver were determined using commercial kits. Values represent the mean ± S.D. from 7 animals. ^∗^*P* < 0.05, ^∗∗^*P* < 0.01, and ^∗∗∗^*P* < 0.001 versus the relative MOD group. BUN: blood urea nitrogen; CON: control; HP: high-dose prescription; LA: lactic acid; LDH: lactate dehydrogenase; LG: liver glycogen; LP: low-dose prescription; MOD: model; PQR: Panacis Quinquefolii Radix.

LA, LDH, BUN, and LG can be used to evaluate the antifatigue activity of drugs. As shown in Figure 2, the induction of weight-bearing exhaustive swimming significantly decreased the LG content in the liver and increased LA and BUN content in the serum. LDH levels in the serum also decreased. These effects were reversed following the administration of the prescription.

### 3.4. Effects of the Prescription on Oxidative Status in Mice

**Figure 3:**
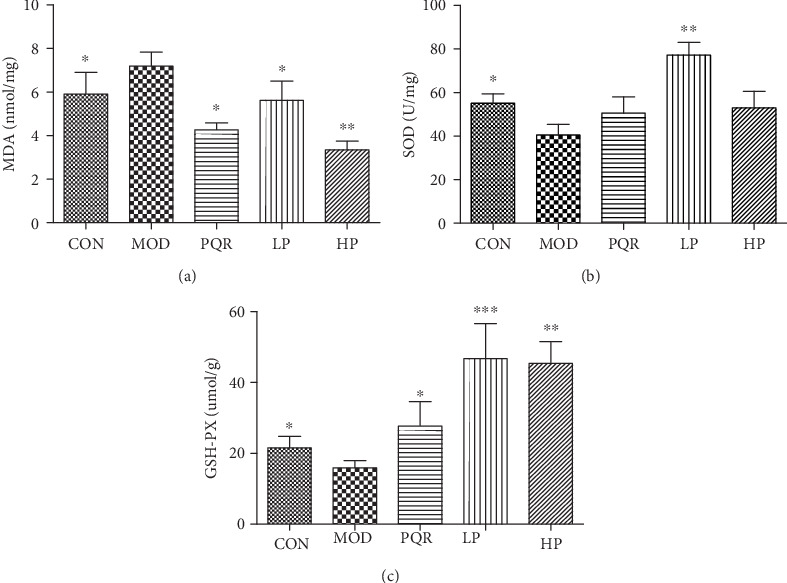
Effects of the prescription on oxidative status in mice. Mice were administered saline, PQR, or the prescription extract (1 or 2 g/kg/day). After 4 weeks of feeding, mice were sacrificed and the livers were collected. Levels of (a) MDA, (b) SOD, and (c) GSH-PX were determined using commercial kits. Values represent the mean ± S.D. from 7 animals. ^∗^*P* < 0.05, ^∗∗^*P* < 0.01, and ^∗∗∗^*P* < 0.001 versus the relative MOD group. CON: control; HP: high-dose prescription; GSH-PX: glutathione peroxidase; LP: low-dose prescription; MDA: malondialdehyde; MOD: model; PQR: Panacis Quinquefolii Radix; SOD: superoxide dismutase.

Oxidative stress is accompanied by fatigue. As shown in Figure 3, MDA levels in the liver significantly increased after weight-bearing swimming in mice, whilst SOD and GSH-PX levels decreased. These effects were reversed following the administration of the prescription.

### 3.5. Effects of the Prescription on Energy Metabolism in Mice

**Figure 4:**
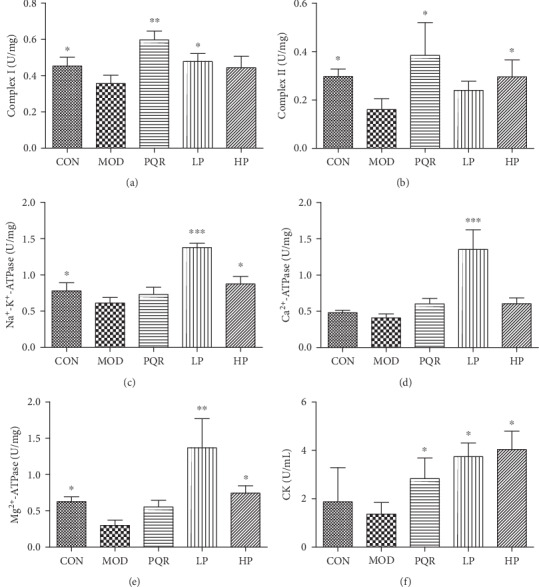
Effects of the prescription on energy metabolism in mice. Mice were administered saline, PQR, or the prescription extract (1 or 2 g/kg/day). After 4 weeks of feeding, mice were sacrificed and serum and hind-leg skeletal muscle tissue were collected. Levels of (a) complex I, (b) complex II, (c) Na^+^-K^+^-ATPase, (d) Ca^2+^-ATPase and (e) Mg^2+^-ATPase in the mitochondria of skeletal muscle, and (f) CK in the serum were determined using commercial kits. Values represent the mean ± S.D. from 7 animals. ^∗^*P* < 0.05, ^∗∗^*P* < 0.01, and ^∗∗∗^*P* < 0.001 versus the relative MOD group. ATPase: ATP synthase; CK: creatine kinase; CON: control; HP: high-dose prescription; LP: low-dose prescription; MOD: model; PQR: Panacis Quinquefolii Radix.

Figure 4 shows that Na^+^-K^+^-ATPase, Mg^2+^-ATPase, and complex I and II levels in the mitochondria of skeletal muscles of hind legs significantly decreased after swimming compared with the NC group. Ca^2+^-ATPase levels in the mitochondria and CK levels in the serum of mice also decreased. However, these effects were abolished by prescription treatment.

## 4. Discussion

The experimental model of weight-bearing exhaustive swimming can be used to study muscle fatigue [8]. In this study, the experimental model was established to study the muscle fatigue-alleviating effects of the prescription, with PQR used as a positive control. Long-term exercise leads to the loss of nutrient, energy, LA, and BUN accumulation but increases free radicals, leading to muscle fatigue [9, 10]. We found that the levels of LA and BUN in the serum significantly increased after swimming, which was accompanied by a loss of LDH and LG. These values were reversed after administration of the prescription, indicating its ability to relieve muscle fatigue.

The relationship between fatigue and oxidative stress is well-defined. When the body is in a state of motion, the balance between the production and elimination of free radicals is disrupted, and free radicals that fail to be eliminated damage human cells, leading to fatigue [11, 12]. The oxidative stress index can be used to evaluate the efficacy of antifatigue therapeutics. We found that weight-bearing exhaustive swimming led to an upregulation of MDA levels and a decrease in SOD and GSH-PX in the livers of mice. However, these effects were significantly reversed by prescription treatment, highlighting its ability to improve free radical scavenging and to delay the occurrence of muscle fatigue.

Fatigue commonly results from excessive energy consumption and an insufficient energy supply for activities. Therefore, the indexes involved in energy metabolism were used to evaluate the antifatigue status [13]. We found that the activity of ATPase and complexes I and II in the mitochondria of skeletal muscle tissue obtained from the hind legs, and CK in the serum were reduced after swimming. These effects were however reversed by the treatment with the prescription, indicating its ability to promote the oxidation rate of the respiratory chain and decrease oxidative phosphorylation uncoupling, further enhancing mitochondrial energy metabolism to alleviate muscle fatigue status.

Furthermore, it is very likely that the improvement in swimming performance may be caused not only by the peripheral effects but also by the brain stimulation effects. Therefore, it is meaningful to use the forced swimming task to evaluate the antidepressant efficacy of the prescription.

## 5. Conclusions

Taken together, our data revealed that the prescription composed of PR and NRR effectively alleviated muscle fatigue status by promoting energy metabolism and antioxidation ability. Thus, the prescription represents a useful drug/nutrient to promote energy metabolism and antioxidation ability, to alleviate muscle fatigue.

## Figures and Tables

**Table 1:** Body weight and food intake of mice.

	CON	MOD	PQR	LP	HP
Initial body weight (g)	38.06 ± 0.92	38.39 ± 1.31	37.83 ± 1.22	37.19 ± 1.37	37.7 ± 1.49
Final body weight (g)	42.59 ± 1.52	43.63 ± 1.94	41.76 ± 2.06	40.79 ± 1.90	41.83 ± 1.65
Body weight gain (g)	4.53 ± 0.59	5.24 ± 0.63	3.93 ± 0.85	3.6 ± 0.53	4.13 ± 0.16
Food intake (g)	1361.10	1456.62	1170.33	1179.62	1300.67

Values represent the mean ± S.D. from 7 animals. ^∗^*P* < 0.05 versus the MOD group. CON: control; HP: high-dose prescription; LP: low-dose prescription; MOD: model; PQR: Panacis Quinquefolii Radix.

## Data Availability

All data used to support the findings of this study are available from the corresponding author upon request.
